# What makes pregnant workers sick: why, when, where and how? An exploratory study in the ready-made garment industry in Bangladesh

**DOI:** 10.1186/s12978-017-0396-0

**Published:** 2017-10-30

**Authors:** Sadika Akhter, Shannon Rutherford, Cordia Chu

**Affiliations:** 1 Centre for Environment and Population Health, Brisbane, Griffith University, 170 Kessels Road, Nathran, Brisbane, QLD 4111 Australia; 2 International Centre for Diarrhoeal Disease Research Bangladesh (icddr,b), Dhaka, Bangladesh

**Keywords:** Female workers, Pregnancy ready-made garment industry, Bangladesh, Hypertensive disorders, Stress, Nature of job

## Abstract

**Background:**

Bangladesh has made significant progress in reducing maternal mortality. Many factors have contributed to this; one is the socio-economic development of the country. The ready-made garment industry is at the forefront of this development creating employment for many women. However, the work environment has the potential to create health problems, particularly for vulnerable groups such as pregnant women. This paper explores perceptions of health problems during pregnancy of factory workers, in this important industry in Bangladesh.

**Methods:**

This study was conducted in four factories using qualitative research methods to provide a view of pregnant workers’ health risks beyond a bio-medical approach. Data was collected through in-depth interviews of pregnant workers and observations of their homes and workplaces. Further, key informant interviews with factory health care providers, government officials and employers revealed different perspectives and experiences. Data was collected in the local language (Bengali), then transcribed and analysed using a framework analysis approach.

**Results:**

Female workers reported that participation in paid work created an opportunity for them to earn money but pregnancy and the nature of the job, including being pressured to meet the production quota, pressure to leave the job because of their pregnancy and withholding of maternity benefits, cause stress, anxiety and may contribute to hypertensive disorders of pregnancy. This was confirmed by factory doctors who suggested that developing hypertensive disorders during pregnancy was influenced by the nature of work and stress. The employers seemed focused on profit and meeting quotas and the health of pregnant workers appeared to be a lower priority. This study found that the government lacks the resources to understand the extent of the problem or the level of compliance with maternity related regulations.

**Conclusions:**

These results indicate the vulnerability of female workers to physical and mental stress at work and associations with their health problems during pregnancy. It identifies the deficiencies of family, workplace and health service support for these pregnant workers, highlighting the urgent need for government and non-government organisations to work with this important export industry to improve health surveillance and monitoring and the enforcement of existing laws to protect the rights and conditions of pregnant women.

## Plain Summary

Globally, 303,000 women died from complications during pregnancy and childbirth in 2015. Most of these deaths took place in developing countries especially in Africa and South Asia. Bangladesh, has made remarkable progress in reducing maternal mortality but in 2014 the maternal mortality ratio was still high at 194 per 100,000 live births. This research was conducted in two cities of Bangladesh through qualitative methods (in-depth interviews, key-informant interviews and observations), to explore the lived experience of pregnant workers in the ready-made garment industry in Bangladesh, including the perceptions of other respondents, regarding the workers’ health problems during pregnancy. Pregnant workers reported that they feel stressed from work and are panicked about losing their job if they cannot meet their production quota due to their pregnancy. They do not have sufficient rest or sleep. The factory doctors reported that pregnant women develop hypertensive disorders due to the nature of their job such as working for long hours in one position, and that work related stress may contribute to their hypertensive disorders during pregnancy. The results of this study should be used by the Ministry of Labour and Employment and other human rights organizations to develop strategies to improve the working environment for pregnant workers to address issues of stress and hypertensive disorders during pregnancy.

## Background

The world has made progress in reducing maternal mortality, yet 830 women die every day due to problems of pregnancy [1]. Ninety-nine percent of maternal deaths take place in low resource countries [2]. Bangladesh has made significant progress in reducing the maternal mortality ratio (MMR) but the most recent figures suggest it remains high at 194 per 100,000 live births in 2014 [3]. Of the direct obstetric causes of death in Bangladesh, hypertensive disorders account for 20% of maternal deaths, the remainder associated with post-partum haemorrhage 31% and indirect causes of death 35% [4]. Ironically, what has enabled this improvement in women’s health is the socio-economic development of the country, which is dependent to a large extent on the work of the women in the ready-made garment factories [5, 6].

Post-war economic expansion increased female participation in paid work with a particularly rapid rise in third world countries [7–9]. In the 1980s Bangladesh started to move from an import-oriented economic policy to an export-orientated one [10]. This export-oriented industrialization policy helped to establish the ready-made garment (RMG) industry and this sector contributes more than 80% of the total export earnings of the country [11]. This industry has provided 4 million people an opportunity to participate in income-earning activities and 80% of them are poor, uneducated women working as factory workers [12–14].

Previous studies conducted with female garment workers found that working in this industry has made women economically independent and empowered [15, 16]. They are the main wage earner for their families and they have some control over their income [17]. Nevertheless, despite their increasing participation in the workforce, little is known about their work and the work environment, nor about how these factors constrain women’s health and reproductive choices [18, 19]. Further, there has been little investigation of these women’s health except from the purely biological and epidemiological point of view [20–23].

When data was collected on the health and safety issues for female workers in the ready-made garment industry for the first author’s (SA) doctoral research, pregnancy, fear of losing their job, stress, and hypertensive disorders during pregnancy emerged as major themes. Limited research has been conducted on the lived experience of pregnant women in the ready-made garment industry in Bangladesh. The aim of this paper is to take a broad perspective of maternal health in the ready-made garment industry in Bangladesh. In order to better understand health problems among pregnant workers this paper explores the economic and social vulnerability of pregnant women, their activities in the workplace and home, the regulatory environment, and how these may affect their health during pregnancy.

## Methods

### Study setting

The study was conducted in two cities of Dhaka district, Mirpur and Savar, in Bangladesh. Most of the RMG factories are established in Dhaka district because the infrastructure is favourable to foreign investors [24]. The population of Dhaka district is 47,424,418 [25]. Twenty five percent of the working population of Dhaka district are garment workers living in slums with very limited infrastructure [26, 27]. Data was collected from December, 2015-July, 2016 in four factories (two from each city) without prior knowledge about workplace conditions at the selected factories. They were chosen on the basis of their export orientation (and hence have compliance conditions imposed by the Bangladesh government and International Labour Organization) and the willingness of factory management to participate [28, 29].

### Study participant recruitment and sampling

A qualitative research approach was employed to collect data. The study participants were selected purposefully to learn in-depth about the issues of the investigation. Six pregnant garment workers were selected for in-depth interviews (IDIs) and 14 key informants (Table 1 presents detail about the types of KII participants) were recruited. All female workers had a minimum of one year work experience in the factory. Pregnant garment workers were contacted through a local NGO in the study area. All of the IDI participants preferred holding the interview at their home. Further key informant interviews (KIIs) were conducted in the offices of the key informants.

**Table 1:** Key informant interview participants (KII) by type

KII Participants by Type	# of participants
*Category 1:* Government officials from the Ministry of Labour and Employment	3
*Category 2:* Supervisors of ready-made garment factory	4
*Category 3:* Doctors working in the ready-made garment factory clinic	4
*Category 4:* Representative from the Bangladesh Garment Manufacturers and Exporters Association (BGMEA)	3
Total	14

### Data collection

IDIs are particularly useful for gaining a detailed understanding of study participants’ experience [30]. The life stories provide information about individual perceptions of life, changes and how the individual copes in a society [31, 32]. We employed individual in-depth interviews including life stories with pregnant garment workers, who had all been diagnosed with hypertensive disorders by factory doctors. Fourteen key informant interviews (KIIs) and 8 direct observations were conducted at the workplace and homes of the IDI participants to address our research questions [33]. Key informant interviews provided our study participants an opportunity to share their expert opinions about the experiences of female workers who suffer from health issues, including hypertensive disorders, during their pregnancy. Interviews with different study participants helped to compare and contrast their opinions to ensure the validity of the data [34]. Prior information was gathered about the key informants. They were contacted directly to arrange interview times.

Open-ended interview guidelines were developed with some initial themes at the beginning of the data collection to guide the interviews. These guidelines evolved during data collection in the field [35, 36]. Some initial themes of the guidelines were to explore a) demographic information of the study participants, b) types of work they do in the work place and home, c) health and safety issues in the work place, d) health services and barriers, e) challenges they face at work during pregnancy. These tentative themes of interview guidelines evolved during field work to understand the lives, experiences, and perspectives of health of the study participants through iterative processes. The length of IDIs averaged from one to one and half hours while KIIs lasted one hour on average. The interviewer (SA) explained the study’s purpose and requested informed written consent from all study participants. All interviews were recorded and detailed field notes were taken simultaneously. The interviews were conducted in Bengali by SA who is a native speaker. Therefore there were no language difficulties in data collection or preparing transcripts. The data was interpreted as much as possible in the original language to minimize the challenge of translation [29].

### Data analysis

After completing each interview, the interviewer (SA) reviewed the field notes and guides to identify emerging themes. In subsequent interviews, probing questions were also added, and the level of data saturation on particular topics was assessed before selecting participants for the next interview. Interviews were then transcribed from audio recordings. Transcripts were checked and compared to the detailed notes to ensure consistency and accuracy. We followed the framework analysis approach to analyse data for this study [37, 38]. All data including interview transcripts and field observations notes were read and re-read line-by-line to develop an open coding on a subsample of interviews in order to finalize a codebook for the framework analysis. These initial codes were synthesized into a coding scheme and applied systematically to all transcripts using Atlas.ti (version 5.2). Finally, existing practices and participants’ perspectives on health problems during pregnancy were analysed for this paper.

## Results

### Characteristics of the study participants

A total of twenty women were interviewed. Out of the twenty women, six of them were pregnant and reported that they had developed health problems, including hypertensive disorders, during pregnancy. They reported that the doctors at the factory clinic had explained these health issues. These women were aged between 20 and 35. None of them had completed primary education (up to grade five). Three of them were working as sewing machine operators: a job that requires sitting constantly in one position on a small stool to run a machine. Three of them worked as a helper, restricted to one position, either sitting or standing, to cut the thread of the fabric. Our interviews with the female workers revealed that the women had migrated from their villages due to poverty to work in the ready-made garment factory. This job created an opportunity for these women to generate income to support their families.

### Health services available to female workers

Female workers can access factory clinics for health care services. The clinics are open from 8 am to 9 pm every day except weekends. They are staffed by nurses and doctors but doctors are available for half days in the afternoon. The female workers explained that they receive painkillers for headache, oral saline for stomach problems, and treatment for injuries (needle puncture, small burn injuries, and cuts from scissors). They indicated that they visit the factory clinics when they get sick from colds or cough, fever and injuries. The clinics also provide ante-natal and post-natal check-ups for pregnant workers.

The women reported that they do not visit the factory doctor for an ante-natal check-up when they first suspect that they are pregnant because they feel they need to hide their pregnancy from their supervisors. The women need to meet a production quota of one hundred pieces per hour. If they lag behind the quota due to their pregnancy, their supervisors will encourage them to leave the job. They will also not be assigned to do overtime to earn extra money. They only go to the factory clinic for a check-up during pregnancy when their pregnancy becomes visible. They also do not go to the private clinics because of the cost.

The women also reported that they cannot go to the government hospital because of their long working hours: *“We enter in the factory at 8:00 am in the morning. We return home at night. We work 10–12 hours per day and sometime 7 days in a week. We need to meet production quota, 100 pieces per hour. I could do it but after getting pregnant I cannot meet my production quota. First few months of my pregnancy, I did not feel well; I felt all time nausea, headache, and fatigue. I took more small breaks and I became slow to meet my daily quota. I did not go to the factory doctor for check-up because my supervisor will know that I am pregnant and would encourage me to leave the job as I was falling behind to meet the target due to my pregnancy.”*

### Factory work, pregnancy and vulnerability

Our interviews with pregnant female workers revealed different perspectives about their health during pregnancy. They reported that the doctor of the factory told them they had a hypertensive disorder, and that “stress” was the “cause” of their health problems. The female workers reported that they didn’t know if there was a relation between stress, work and illness. They reported that getting pregnant is a ‘normal’ experience that ‘every’ woman goes through, but the experience of pregnancy made them less able to cope with their work due to the pressure and the nature of the job.

The following stories of two pregnant women who work in the ready-made garment industry explore how the nature of their job, and work practices at home and in the work place affect their health. The narratives are from two women, “Afia” and “Morium” (both pseudonyms), who had developed hypertensive disorders during their pregnancy as described to them by the factory doctor. Participant quotes are presented to support findings.

#### Afia’s story

Afia was a twenty year-old woman from Gaibandha district in the northern part of Bangladesh. She came to Dhaka for a job at the garment factory with another woman who lives in the same village. The woman helped her to get a job as a machine operator. She has been working in the factory for the last three years. At the time of the interview, she was seven months pregnant. Her husband is a driver. She was married three years earlier and she had been experiencing pressure from her in-laws family to have a child. Three years work in the garment factory taught her to avoid getting pregnant, as the cost of pregnancy would be losing her job. According to her, she got pregnant accidently and she wanted to have an abortion but her husband said that if she had an abortion he would divorce her. She continued with her pregnancy. She said that around the fourth month of her pregnancy, one day she got so sick, that she couldn’t work, and she couldn’t talk, so her co-workers took her to the factory clinic. The doctor of the factory clinic said that she was suffering from high blood pressure. He advised her to take a rest during work and that she shouldn’t work for long hours and she also needed to sleep well. On that day she wanted to have some time off but her supervisor refused her request for time off as a large order of shirts had to be finished in fourteen days. She needed to complete 100 shirts per hour, for 12 hours a day, until the order was complete. In her seventh month, she informed her line manager that she would apply for maternity leave as her blood pressure was always high and she always felt sick. Her supervisor suggested that she leave the job and come back to work after the delivery.

This job is her only source of income. She needed the money, so she spent sleepless nights worrying: *“This work created an opportunity for me to earn money. I could support my family financially but now I am pregnant. When we get pregnant we don’t disclose it to our supervisors. We try to keep it secret as long as we can because if we become slow to meet our production quota due to pregnancy we will lose our job. We become more vulnerable physically and mentally due to all these types of stress.”*

#### Morium’s story

Morium is a 28 year-old woman who developed a hypertensive disorder during pregnancy. She always experienced headache, nausea and dizziness. She did not go to a doctor as she believed that these symptoms were normal during pregnancy. After a few weeks she started to feel sicker and couldn’t sit for work and couldn’t sleep well. She then went to the factory clinic to see the nurse. The nurse informed her that her blood pressure was high. The nurse also told her not to work and that she should go home. After three days she visited the clinic again and she was able to see the factory doctor. The doctor advised her not to eat salt or beef and that she needed to sleep more. The doctor recommended that she take medical leave for three days with some medication. She was granted sick leave for just one day. After two days she returned to her work but she always felt unwell. At five months pregnancy, her legs and face become swollen (oedema). She constantly feels tired, short of breath and pain in her shoulders. Still she wants to continue her job as long as she can. She will apply for maternity leave. If the leave is granted with payment she will continue the job after delivery, if not she will leave the job and will look for a new job when the child is 6 months old.

### Their living environment: Opportunities to rest at home and work

**Fig. 1:**
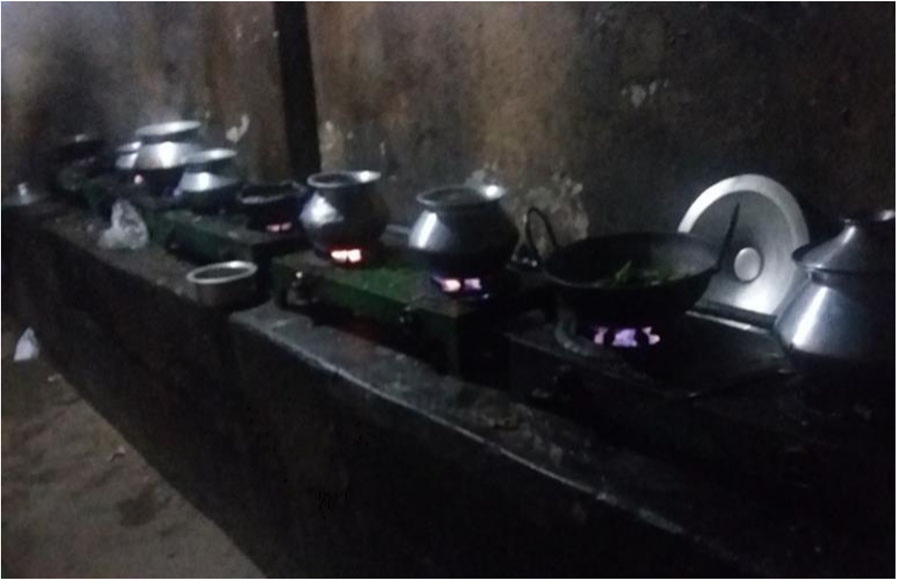
Long queue in the kitchen to cook

The houses of the female workers were observed in two study areas. After walking through a narrow lane, the researcher entered a one level brick and tin building with twenty single rooms (one family, two persons in each room, the size of the room is 30–40 square feet), one shared kitchen with some stoves for cooking (Fig. 1), and four toilets for twenty families. The furniture includes a double bed which occupies most of the space of the room. The clothes are hung on a rope; cooking utensils are piled under the beds. The rooms do not have window, only a door to let in air and light. The female garment workers start their day around 4:00 am to avoid queues in the kitchen and toilets. They need to finish cooking for themselves and their family members, cleaning and other household chores before they go to the factory. The women’s living environment also worsens their health condition because shared accommodation does not allow them to have enough sleep or rest at home.

In this regard one female worker said, *“My doctor said to me to sleep more. How could I sleep more? I work for long hours and have little time to prepare meals. I return home tired every day. I just eat rice with some vegetables, dal and salt before I go to sleep. I cook fish and meat sometimes but that I keep for my husband.”*

**Fig. 2:**
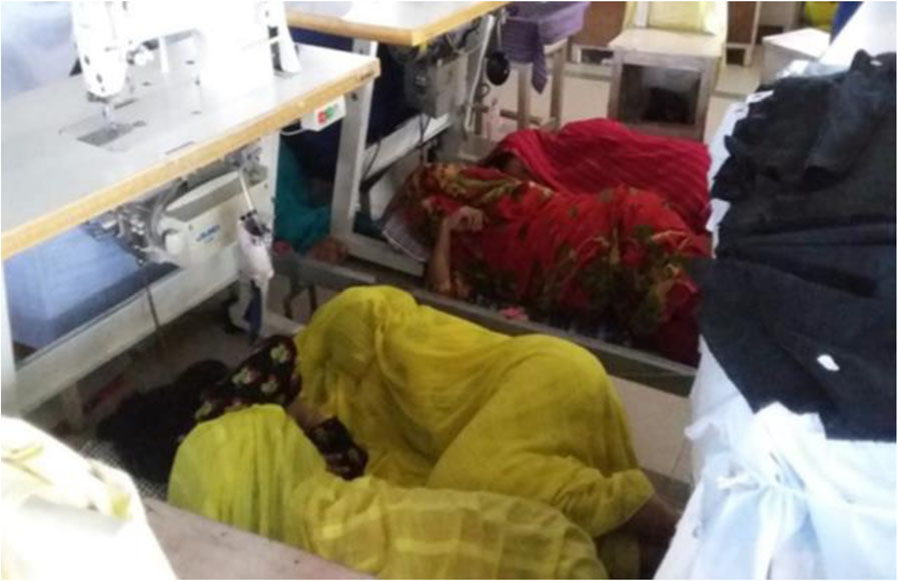
Little rest during work in the lunch break

They work hard at home in the morning and walk to the factory as fast as they can to enter the factory by 8:00 am. They get a one-hour break for lunch. The pregnant workers consume food very quickly as to take a small nap during lunch break (Fig. 2) to energize them for the rest of the workday.

Reflecting on the experience of pregnancy and work, another pregnant worker noted:

*“Pregnancy is the most joyful event for a woman. [But] if you work as a factory worker, it will be the most painful event of your life. Working in one position for long hours, running to meet the production target and having no guarantee to have maternity leave with payment makes us sick. If you are pregnant you may have to lose your job. Supervisors will not be happy with you as you will be slow to meet the daily quota. You will be encouraged to leave the job. You will not receive support from your boss to continue the job as a pregnant woman. The doctor will advise you to take rest but you will not have an opportunity to take rest due to work at home and workplace.”*

### Factors that influence the development of health problems during pregnancy

The factory doctors reported that the work stress and prolonged working hours contributed to hypertensive disorders and diabetes among the pregnant workers. Others factors such as biological condition and lifestyle also contribute to making women sick: *“the female garment workers work for long hours and they always need to work to meet their daily production quota. When women get pregnant it is difficult for them to meet the production quota. They always feel stress because if they cannot meet the production quota they will lose their job, which definitely contributes to increasing their blood pressure.”*

Another doctor said, *“If you look at their lifestyle they do not have time to rest. They work in the factory 10–12 hours per day 6 days in a week. Most of the female workers develop hypertensive disorders during pregnancy. Apart from hypertensive disorders, they also suffer from other health problems such as malnutrition and anaemia. They couldn’t eat well due to poverty. We always advise them to eat an egg and not to take any extra salt with rice. They cannot continue their job during pregnancy because this is a physically intensive job. They take a break for one to two years after the delivery, and then when the child grows up they again start a new job with a new factory.”*

### Business, profit and workers’ health

Discussion was held with the employers of the ready-made garment industry to explore their views on the health problems including hypertensive disorders among the pregnant workers. They reported that pregnancy makes the women more vulnerable in this sector due to the nature of the job. They stated that they always try to allow more breaks for pregnant women, but they cannot always ensure maternity leave with payment.

In this regard one supervisor said, *“We do business. We need to ensure profit. If we cannot make profit, we will not be able to pay the workers. We have hundreds of workers working on the production floor. If we allow maternity leave with payment, hundreds of them will get pregnant. How will the factory run? It does not mean that we do not provide maternity leave with payment. We approve maternity leave with payment. [But] in some cases we also cannot approve leave with payment. Because many of these women they go to their village for delivery. They take the money from us but they do not return to work after their leave. In these cases we encourage women to leave the job but if they want to return to their work after delivery, we accept them again in the work.”*

### Issues of inadequate reporting and non-compliance with maternity legislation

Interviews with government officials from the Ministry of Labour and Employment revealed that they do not have enough reliable data about the health and injury related problems of the factory workers because they are short of skilled staff to collect data on occupational health and safety issues. They also indicated that the factories do not report pregnancy or pregnancy-related health problems to them for fear of being followed up by government factory inspectors.

In this regard one respondent said, *“When our factory inspectors visit factories they always request the factory management to assign light work for the pregnant workers. They also instruct them to approve maternity leave with payment. According to the Maternity Act of Bangladesh, if any worker is found to be pregnant, the factory should report that to us, but the factories do not report because they need to approve their maternity leave with payment. As they do not pay them, so they do not report to us about the pregnancy. We cannot tell you about the health problems of these women. We lack reliable data.”*

Another respondent raised the important challenge of non-compliance with existing legislation and the concerns expressed by women about this lack of compliance: *“We receive many complaints of disputes about maternity benefit. The factories do not want to approve maternity leave with payment for pregnant women. According to law, they need to pay 60 days leave before delivery and the rest of the leave after delivery. In most of the cases they approve leave without payment. In some cases they approve leave with maternity benefit for 6o days but they do not pay for the other sixty days.”*

## Discussion

This study found that the factory work in this industry is physically demanding and stressful for the female workers who have long working hours, live with constant fear of dismissal as well as significant family and societal demands on their time at home. During pregnancy, their vulnerability to these issues increases. It further revealed that pregnant workers in these factories work in one position or stand for long periods of time with limited or no breaks. Some studies have found that such physically demanding work with prolonged standing and long working hours can increase catecholamine levels, and patients who have high levels of catecholamine suffer from pre-eclampsia or hypertensive disorder [39, 40]. Though the workers in this study had no formal diagnosis of high levels of catecholamine, their working conditions paralleled the conditions of these other studies and factory doctors indicated that pregnant factory workers commonly suffered from health problems such as hypertension.

Antenatal care (ANC) is identified as one of the four pillars under the safe motherhood initiative and seeking care during pregnancy and delivery is crucial to protect the women from complications related to pregnancy and childbirth [41]. Other studies have found that inadequate provision of ANC is one of the risk factors for maternal morbidity and mortality [42, 43]. This study clearly identified a deficiency in access to quality antenatal care. While there are health services available within the factory (half-time doctors and full-time nurses), women are reluctant to seek ANC services from them for fear of losing their jobs. Further, these women work very long hours (10–12 hours per day and 6 days, sometimes 7 days per week) and have little time or cannot afford to access external (to the factory) health services.

Another key finding of this study was the constant stress related to meeting production quotas and hence making sufficient income. This is particularly problematic for the pregnant women as they are unable to rest and constantly fear they will lose their job due to their pregnancy. The women have low decision making power in their work life, and they do not receive institutional support. All these factors have interconnections with the health and illness of pregnant workers. The findings of this study parallel the job stress model which focuses on the work environment, including job demands or workload, and the scope of decision making for a worker in performing her work [44]. The lack of institutional support identified in this study includes lack of access to formal maternity leave, lack of job task modifications during pregnancy (eg. light duties) and lack of increased breaks and time off for health care or rest. Government informants acknowledged this and their limitations in responding to this problem while factory managers, although they claimed to provide these supports, acknowledged that the pressures to meet quotas and make profit dominated their management of their workforce.

These findings indicate some specific areas for action. First, study findings should be communicated to key stakeholders including the Ministry of Health, Ministry of Women and Children Affairs, Ministry of Labour and Employment, BGMEA, factory owners and development partners to facilitate dialogue for developing interventions to create awareness about, and prevent health risks associated with, pregnancy and work in the sector. More broadly, the results suggest that coordinated action and commitment needs to be taken by key stakeholders to create an enabling environment and gender sensitive policies for working women so that they can participate in paid work and be treated fairly particularly during vulnerable times such as pregnancy.

Second, essential maternal care comprises antenatal, delivery and postnatal care to prevent maternal morbidity and deaths [45]. Women in this study did not access factory health services for antenatal care for fear of losing their job and they indicated difficulties in accessing ANC services from government hospitals. This requires an awareness-raising intervention targeting women, their husbands and workplace supervisors of the importance of ANC to pregnancy outcomes. As factory clinics are an existing and easy to access service for these women, this could be the focus for this type of program, however issues of confidentiality and independence of factory health staff need to be resolved.

Third, the BGMEA, factory owners, the government and the buyers need to work together to improve processes to ensure that maternity benefits are provided to those who are eligible to enhance the job security of pregnant workers. These findings suggest a need for better implementation of the national maternity leave policy for pregnant workers in order to ensure their maternal health and job security. Further, changes in work organisation need to be considered by the industry to help to reduce physical job stress in pregnant working women by altering work hours, improving rest opportunities and rotating job tasks.

Fourth, global buyers, researchers, advocacy groups and other key stakeholders must collaborate to develop strategies to address the issue of a buyer driven production system, stress and its impact on the health of the workers in this industry. It is clear from these findings that profit and lack of regulatory monitoring are contributing to health problems for this important part of the industry’s workforce.

### Strength and limitations

The strength of this study is that it provides an opportunity for women, employers, government regulators and the industry peak body (BGMEA) to explore health issues for pregnant workers in this important industry in Bangladesh. Multiple voices allowed for triangulation of key findings, particularly issues relating to health and potential for hypertensive disorders during pregnancy, working environment and stress.

However, the findings of this study should be interpreted with caution as it was not designed to test a hypothesis and hence cannot provide quantitative links between work, pregnancy and hypertensive disorders in this industrial sector. Further, as interviews were conducted with pregnant women who had little or no education, their reproductive knowledge may be poor and hence their narrative, particularly about timing of pregnancy and illness during pregnancy may suffer from recall bias.

This study presents findings from a small number of study participants in four ready-made garment factories in Dhaka, Bangladesh. The findings of this research may not reflect the experiences of pregnant workers across the whole ready-made garment industry in Bangladesh.

## Conclusions

This study describes the lived experience of pregnant workers in the ready-made garment industry in Bangladesh based on their own narratives. The findings of this study highlight the broader social and economic context of illness to extend our understanding of women’s health beyond conventional ways of exploring women’s health. The pregnant workers’ working conditions, their position in the factory, their living environment, and their roles at home all contribute to their health problems and illness during pregnancy. The results of the study are similar to other studies that have investigated the links between the nature of jobs, the social class of the female workers and stress [46, 47].

Pregnancy is an important time in a women’s life. This study explores how multiple factors can affect female garment workers’ health during pregnancy. Despite the importance of the female workforce to the growth of this industry and its consequent positive contribution to Bangladesh’s economy, this study indicates that more needs to be done to protect the health of pregnant workers working in the industry. Key issues of access to antenatal care, modification of workplace conditions during pregnancy, lack of institutional and societal understanding and support about work during pregnancy, and enforcement of maternity leave regulations require urgent government and industry commitment and collaboration.

## Abbreviations

ANC: Ante-natal care
BGMEA: Bangladesh Garment Manufacturers and Exporters Association
GU: Griffith University
ICDDR, B: International Centre for Diarrhoeal Disease Research, Bangladesh
MMR: Maternal Mortality Ratio
RMG: Ready-made Garment
